# Aggressive vascularized odontogenic myxoma. Case report and literature review

**DOI:** 10.1016/j.ijscr.2025.111060

**Published:** 2025-02-14

**Authors:** Luis Pacheco-Ojeda, Miriam Díaz-Yépez, Germán Castillo-Aguirre, Luis Mogrovejo-Freire, Stalin Cañizares-Quisiguiña

**Affiliations:** a Surgery Service, Hospital Metropolitano, Quito, Ecuador; b Pathology Service, Clinica Novaclínica, Quito, Ecuador; c Interventional Radiology, Clínica Harvard, Quito, Ecuador; d Otorhinolaryngology Service, Hospital Pediátrico Baca Ortiz, Quito, Ecuador; e Universidad San Francisco, Quito, Ecuador

**Keywords:** Odontogenic myxoma upper jaw

## Abstract

**Introduction and importance:**

Odontogenic myxoma (OM) is an infrequent benign mesenchymal odontogenic tumor of the jaws composed of rounded and angular cells dispersed in an abundant mucoid stroma. An aggressive and vascularized presentation is unusual.

**Case presentation:**

A 68-year-old woman was evaluated for a mass located under the palate and extended to the cheek. A large flattened mass covered the entire palate, except the left lateral dental arch, and extended to the cheek as a firmed 6 × 6 × 5 cm mass. An enhancement computed tomography and a magnetic resonance imaging revealed a large, heterogenous, expansive, 80 × 64 × 58 mm mass that destroyed the right maxilla and extended to the soft tissues of the cheek. Minimally invasive endovascular angiography and embolization was performed preoperatively. Through a modified Weber-Ferguson incision, a partial right lateral maxillectomy was performed, conserving the right nasal fossa and a left canine tooth, medially, and the floor of the orbit, upward. Pathology study reported an odontogenic myxoma with compromised margins but reexcision margins were negative. One year after surgery, there is no evidence of disease.

**Clinical discussion:**

Pathological and radiological differential diagnosis that includes a large number of benign and malignant lesions are discussed. All authors agree that wide surgical excision is the treatment of choice.

**Conclusions:**

Wide excision resulted in good functional and local control. Preoperative embolization is needed in case of a vascularized lesion. No immediate surgical reconstruction should be recommended to facilitate clinical surveillance and early recurrence detection.

## Highlights

• Highly vascularized odontogenic myxomas have not been mentioned in large series.
• This type of lesions may require preoperative arterial embolization to reduce bleeding.
• Locally aggressive odontogenic myxomas are uncommon.
• Wide excision is the treatment of choice and results in good functional and local control.

## 1 Introduction

Odontogenic myxoma (OM) is an infrequent benign mesenchymal odontogenic tumor of the jaws composed of rounded and angular cells dispersed in mucoid stroma. Its histogenesis seems to be related to the odontogenic ectomesenchyme of a developing tooth [1,2]. OM which was classified as myxofbroma in the 2017 World Health Organization Classification of Head and Neck tumors, is now categorized under fibromyxoma in the 2022 5th edition [3]. OM described incidence has ranged from 0 to 18.3 % among odontogenic tumors [4,5]. We describe the case of a 68-year-old woman who developed an aggressive and vascularized odontogenic myxoma of the upper jaw, gum and hard palate. We have reviewed the literature about the epidemiology, clinical and pathological features, differential diagnosis, and treatment modalities of this rare tumor.

This case is reported according to the SCARE criteria [6].

## 2 Case report

**Fig. 1:**
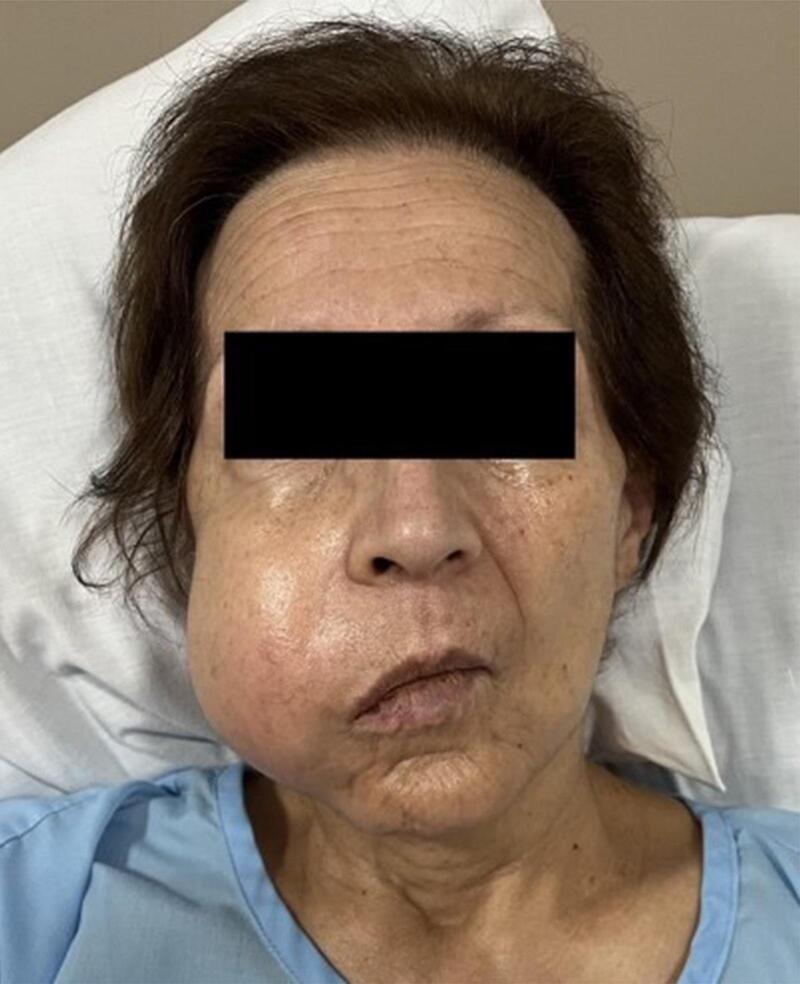
Appearance of the face of the patient.

**Fig. 2:**
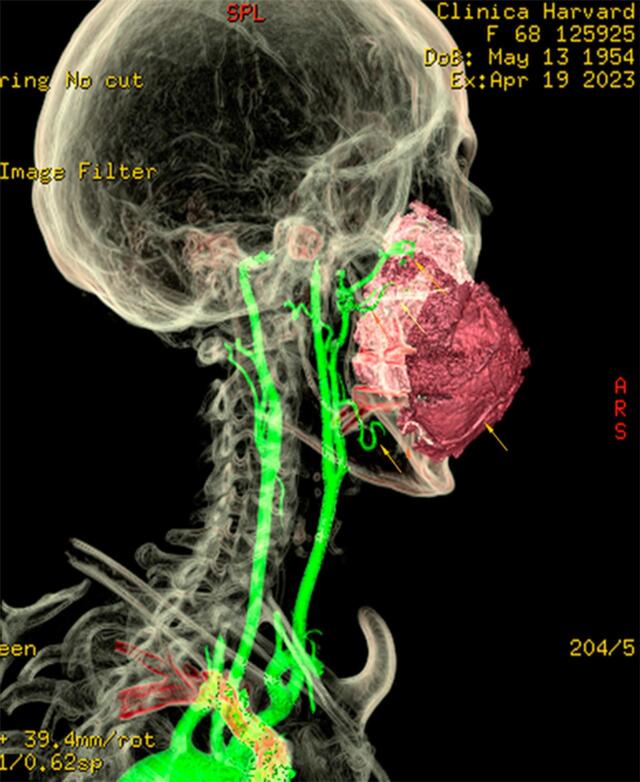
Vascular 3D reconstruction demonstrates moderate tumoral perfusion from the right facial and internal maxillary arteries.

**Fig. 3:**
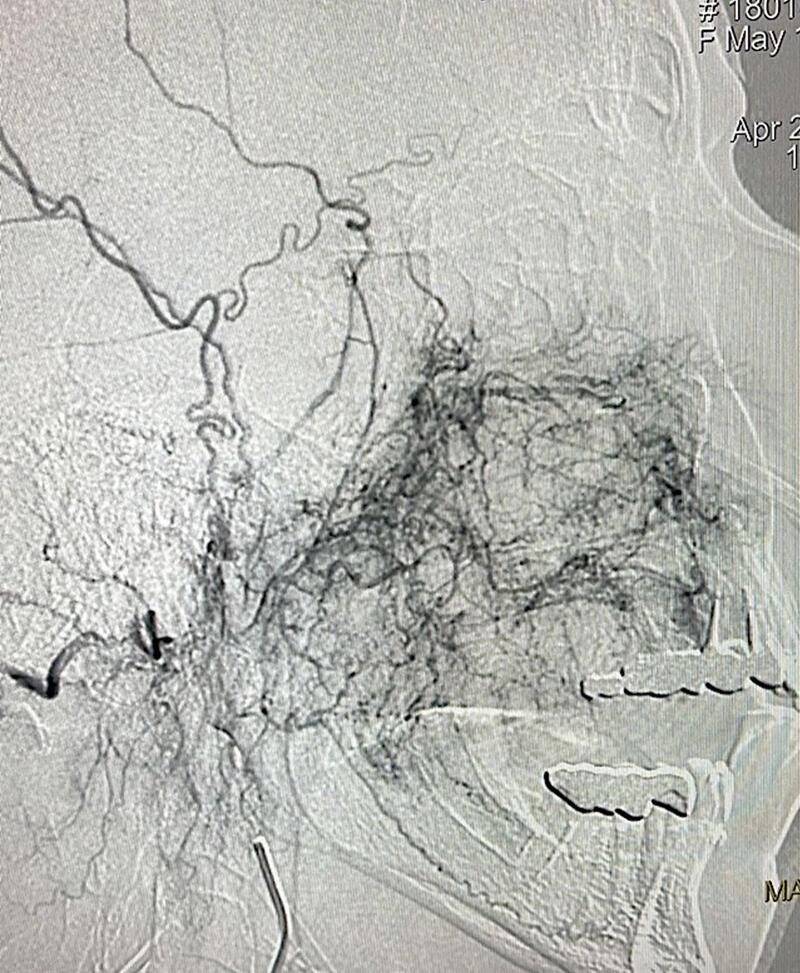
Pre-embolization diffuse subtraction angiography (DSA) of right internal maxillary and facial arteries and post-embolization.

A 68-year-old otherwise healthy woman was clinically evaluated for a mass located under the palate and extended to the right cheek. A mass had grown at the right upper gum adjacent hard palate three months after extracting a molar tooth. Biopsy revealed an angiomyxoma. On physical examination, a large flattened mass covered the entire palate, except the anterior and left lateral dental arch, and extended to the cheek as a firmed 6 × 6 × 5 cm mass (Fig. 1). A magnetic resonance imaging (MRI) revealed a large, heterogenous, expansive 80 × 64 × 58 mass that destroyed the right maxilla but respected the floor of the orbit. Enhancement computed tomography (CT) showed disruption and cortical erosion of the right maxillary bone (alveolar septa, zygomatic process) by a 8.4 × 7.6 cm tumor that extended toward the oral cavity and the soft tissues of the cheek. Soft tissue image demonstrated a well-defined unilocular homogeneous mass. The hypodense tumor measure ranged from 45 to 111 Hounsfield units including soft tissue with and without vascularized areas. Vascular 3D reconstruction demonstrated moderate tumor perfusion from the right facial, and the internal maxillary arteries (Fig. 2). Minimally invasive endovascular angiography and embolization of the aforementioned arteries was performed 3 days before surgery. We used 500–700 and 700–1000 μm PVA particles through a vertebral F4 catheter to occlude the arteries, achieving total embolization (Fig. 3).

**Fig. 4:**
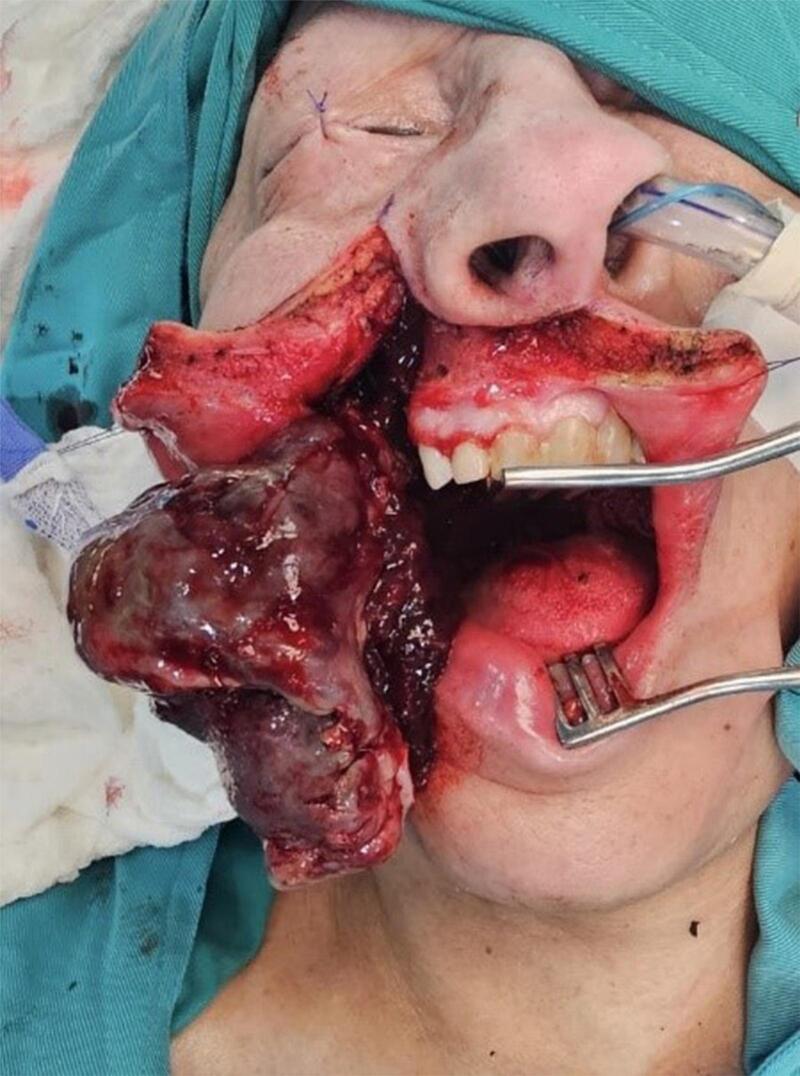
Modified Weber-Ferguson surgical approach.

The patient signed an informed consent before surgery. Through a modified Weber-Ferguson incision, to adequately expose the tumor, a partial right lateral maxillectomy was performed, conserving the right nasal fossa and the left canine tooth, medially, and the floor of the orbit, upward, that appeared free of tumor (Fig. 4). Bleeding was minimal.

**Fig. 5:**
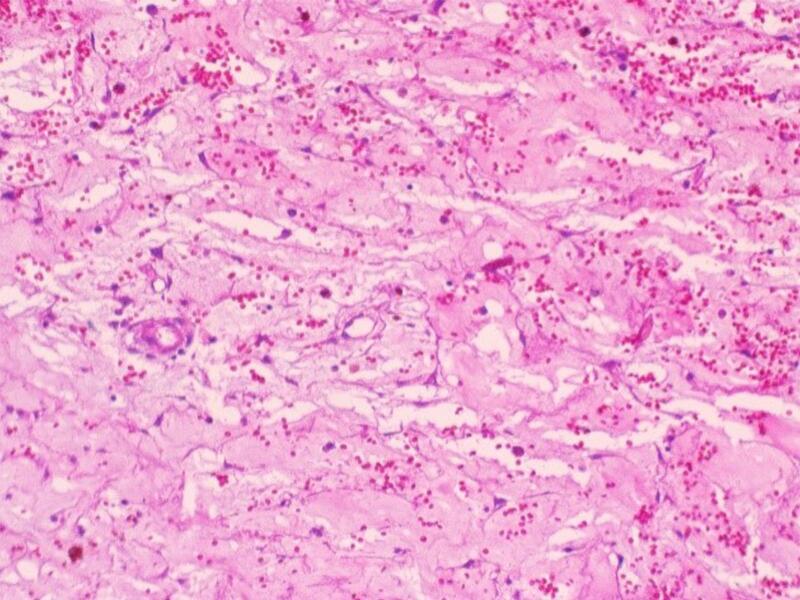
Few spindle cells scattered in abundant myxoid stroma. Abundant vascularization to highlight.

**Fig. 6:**
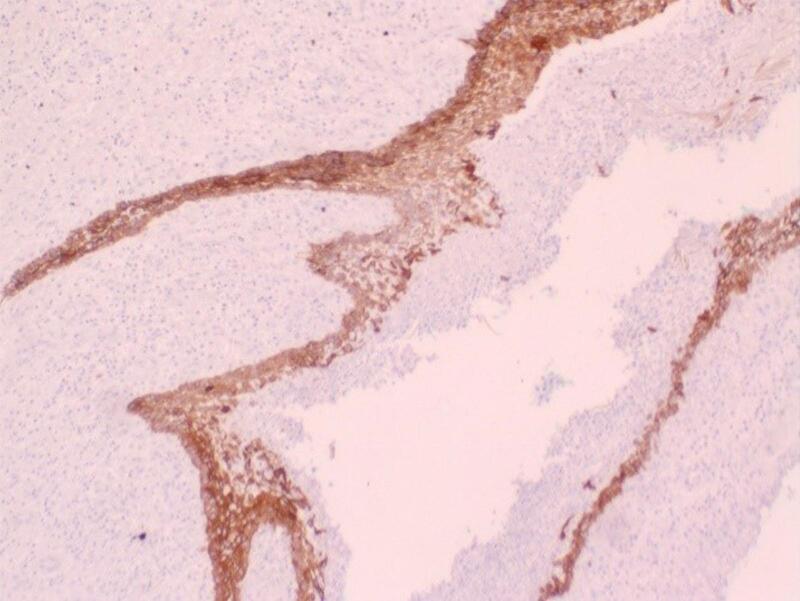
Immunohistochemistry (IHC) Cytokeratin 5/6 positive in squamous epithelium corresponding to cell rests of Malassez.

An 8.7 × 6 × 4.5 cm soft tumor mass was received for histologic study. It weighed 97 g and presented a poorly defined external surface. It was bounded by mucosa on one side and by a gray-red lobulated, myxoid and red surface on the other. Additional dark brown fragments measuring 11 × 5 × 1 cm in total were also received. Histopathological examination revealed uninucleate, spindle-shaped and stellate cells dispersed in a background of abundant mesenchyme. Cells had nuclei that did not show any abnormality or variation in number or structure and long cytoplasmic processes at either end. Abundant lymphoplasmocytic and polymorphonuclear infiltrate was appreciated. Copious gross and dilated arterial and capillary vessels occupied a significant area around which a neoplastic nest was observed. The neoplastic area extended toward medullary spaces between bone spicules and ciliated pseudostratified cylindrical respiratory epithelium. Newly formed thrombosis was also detected inside these vessels, as well as occasional hemorrhagic areas. Immunohistochemistry showed epithelial cell rests of Malassez (positive for CK5) and positive Ki67 up to 9 %. The pathology report concluded an odontogenic myxoma (Fig. 5, Fig. 6) with chronic active inflammation and compromised surgical margins. A reexcision was performed two weeks later and the pathological study of the buccal mucosa, the maxillary bone and maxillary sinus mucosa margins were negative.

Postoperative evolution was uneventful and a palatal prosthesis was placed postoperatively. One year after surgery, there is no evidence of disease and, speech and deglutition are satisfactory.

## 3 Discussion

Myxomas are very rare benign, locally invasive tumors, of mesenchymal origin. They occur in hard sclerous and soft tissues of the body such as the skin, bones, subcutaneous tissue, skeletal muscle, heart and others. Chrcanovic [1] published in 2018 an updated analysis of 1.692 cases based in 377 publications of series and case reports, and Uchoa-Vasconcelos [2] reviewed 30 published series, with 999 cases in 2018, including a Brazilian series of 85 cases in 63 years.

Our patient was older than reported; from 10 to 40 years (mean 28.6), with a peak in the third decade of life [1,2,4]. There is no specific gender predominance [1,2]. There appears to exist a light predilection for the mandible as the site of origin of the tumor [1,4]. It has been described that the posterior region of the maxilla, usually a premolar or molar, as in our patient, would be the site of origin of the tumor in 19 to 41.1 % of cases [4,5,7].

The OM is composed by bland myxoid cells, without atypia, arranged randomly with dilated capillaries [8,9]. It has an invasive behavior into the neighboring bone, and this seems to be due to the expression of matrix metalloproteinases 2 and 9 that degrade the extracellular matrix, favor the entrance of tumor cells into the bone trabeculae, and consequently tumor growth [9]. The presence of abundant dilated arterial and capillary vessels, as in our case, is unusual.

The microscopic differential diagnosis for OM includes, more commonly, normal dental papilla, hyperplastic dental follicle, aggressive angiomyxoma, angiomyofifbroblastoma, myxoid neurofibroma, chondromyxoid fibroma, odontogenic fibroma and low-grade fibromyxoid sarcoma [3,10].

The aggressive angiomyxoma and angiomyofibroblastoma contain more cellular diversity including capillaries and thin-walled hyalinized vessels that surround hemorrhagic areas in conjunction with collagen fibers, fusiform cells and some perivascular epithelioid cells, and are usually localized in the genital region and the retroperitoneum or pelvis. They are positive for vimentin, desmin, estrogen, progesterone and, eventually, CD34+, but usually negative for S100 [11].

Some neoplasms such as myxoid neurofibroma can also present myxoid areas, but they tend to present a different vascular disposition and would most likely contain neurons that are S100(+). Myxoid leiomyoma, located mostly in the pelvis, contains muscular fibers and vessels in a different organization. Among malignant neoplasms that contain myxoid structures, fibromyxoid sarcoma and liposarcoma should be considered [8].

Tumor size of our patient was 8 cm, larger than mean size, 4.7 cm (0.5–25), described in Chrcanovic‘s review [1]. The tumor may be an incidental finding. In most cases, these tumors are asymptomatic as in our patient, but in 17 % of cases, they may cause symptoms such as pain and paresthesia [1,7]. Tooth displacement and mobility, as in our patient, occurred in 54 % of cases in Chrcanovic‘s review [1]. Swelling and bone expansion are very common and variable depending on tumor extension. In general, the clinical behavior is characterized by imprecise symptoms, aggressive behavior and frequent recurrences.

Radiographically, OM appearance may vary from a unilocular radiolucency to a multilocular lesion, with well-developed locules, consisting of fine trabeculae, arranged at right angles, with a well-defined or diffuse margins [12]. Theses tumors are located in the mandible in two-thirds of cases and in the maxilla one-third.

Conventional radiography includes panoramic radiographs and occlusals. The radiological features to be considered include location, appearance of the internal structures, locularity, expansion, margins of the tumor, and the association with unerupted or impacted teeth. In one series [2], the lesions appeared multilocular radiolucent in 61.5 % of cases, unilocular radiolucent in 34.5 %, and in 4 % had a mixed appearance.

Both CT and MRI are highly accurate [12]. From 50 to 76,9 % the tumors appear hypodense compared with muscle, whereas in the remainder they are isodense [13]. CT demonstrates soft tissue invasion, focal interruption, and absence of the smooth pseudocapsule. It allows 3-D modeling, valuable to assess the extent and plan reconstructive procedures [[13], [14], [15]].

Highly vascularized OMs have not been described in large series [1,2]. They may require embolization to reduce bleeding. Arterial embolization followed by a complete non-mutilating resection, results in good functional and local control as in our patient.

Differential radiological diagnoses include ameloblastoma, intraosseous hemangioma, aneurysmal bone cyst, glandular odontogenic cyst, central giant cell granuloma, cherubism, metastatic tumor, simple cysts, odontogenic keratocyst, calcifying epithelial odontogenic tumor, myxoid neurofibroma, myxoid lipoma, chondromyxoid fibroma and osteosarcoma [7].

Wide surgical excision with no less than 2 mm free bone margins, is the treatment of choice. It must be complete, even if the tumor invades deep planes. Curettage and enucleation, eventually followed by peripheral osteotomy, have been other performed surgical modalities. Smaller lesions may be treated by enucleation and larger lesions by segmental resection. Surveillance, debulking or radiation therapy were used in only 6 of the 1592 cases of Chrcanovic‘s review [1]. This type of tumor is not radiosensitive.

OM may have a high rate of recurrence. After a mean follow up of 49 months, recurrence occurred in 9.3 % of cases in Chrcanovic‘s review [1]. It varied according to the type of surgical technique used for tumor removal: 31.3 % after curettage, 13.1 % after enucleation, 12.7 % after enucleation + curettage, 6.7 % after enucleation + peripheral osteotomy, 1.3 % after marginal resection and 3.1 % after segmental resection. Other variables, such as locularity, jaw site or bone expansion, do not seem to have significant influence on recurrence rate [1]. Free microvascular flaps such as the vascularized free fibular flap, have been described for the reconstruction of segmental mandibular defects [16]. To decide a reconstruction, the possibility of an undetected early local recurrence must be taken in account so it should be delayed until an adequate follow up is obtained. In our patient, age was another factor for not performing an immediate microvascular reconstruction.

## 4 Conclusions

Wide excision of odontogenic myxomas as performed in this aggressive and vascularized OM is the gold standard of treatment. Preoperative embolization is needed in case of a vascularized lesion. No immediate surgical reconstruction should be recommended to facilitate clinical surveillance and early recurrence detection.

## Author contribution

Conceptualization; Data curation; Formal analysis; Funding acquisition; Investigation; Methodology; Supervision; Validation; Visualization: Pacheco-Ojeda.

Writing - original draft: Pacheco-Ojeda, Mogrovejo-Freire.

Writing review & editing: Castillo-Aguirre, Cañizares-Quisiguiña.

## Consent section

Written informed consent was obtained from the patient for publication of this case report and accompanying images. A copy of the written consent is available for review by the Editor-in-Chief of this journal on request.

## Ethical approval

Ethical approval was obtained from the Ethics Committee of the Batan Clinic and Medical Center with a reference number 2024-01 on October 10th, 2024.

## Guarantor

Luis Pacheco-Ojeda, MD.

## Research registration number

None.

## Sources of funding

The authors declare that they have not had any funding support.

## Declaration of competing interest

The authors declare that they have no known competing financial interests or personal relationships that could have appeared to influence the work reported in this paper.
